# *CLOCK 3111TT* Genotype Is Associated with Increased Total Cholesterol and Low-Density Lipoprotein Levels in Menopausal Women with a Body Mass Index of at Least 25 kg/m^2^

**DOI:** 10.3390/pathophysiology28010001

**Published:** 2020-12-30

**Authors:** Natalya Semenova, Irina Madaeva, Sergey Kolesnikov, Lyubov Rychkova, Tatjana Bairova, Marina Darenskaya, Lyubov Kolesnikova

**Affiliations:** Scientific Centre for Family Health and Human Reproduction Problems, 664003 Irkutsk, Russia; nightchild@mail.ru (I.M.); sikolesnikov2012@gmail.com (S.K.); rychkova.nc@gmail.com (L.R.); tbairova38@mail.ru (T.B.); marina_darenskaya@inbox.ru (M.D.); kolesnikova20121@mail.ru (L.K.)

**Keywords:** *CLOCK* 3111T/C polymorphism, atherogenic lipids, body mass index

## Abstract

Lipid profile comparative analysis was performed to reveal the interdependence of lipids with *Circadian locomoter output cycles protein kaput* (*CLOCK*) 3111T/C gene polymorphism in menopausal women with/without a body mass index (BMI) of ≥25 kg/m^2^. Methods: A total of 193 female volunteers aged 45 to 60 years were divided into two groups: Those with BMI < 25 kg/m^2^ (control) and those with BMI ≥ 25 kg/m^2^. Each group was then divided into two subgroups: Those with the *CLOCK TT*-genotype and those with the *CLOCK TC-*, *CC*-genotypes. Lipid metabolism parameters were determined by the enzymatic method. Single-nucleotide polymorphisms (SNPs) were detected via polymerase chain reaction–restriction fragment length polymorphism technology. Results: There were no differences in *CLOCK 3111T/C* genotypes or allele frequency between the control and main groups. In addition, there were no differences in lipid profile parameters between women of the control group and different *CLOCK 3111T/C* genotypes. The total cholesterol (*p* = 0.041) and low-density lipoprotein cholesterol (*p* = 0.036) levels were higher in the subgroup of women with a BMI ≥ 25 kg/m^2^ and *CLOCK TT*-genotype as compared to the subgroup with a BMI ≥ 25 kg/m^2^ and minor allele *3111C.* Conclusions: SNP *3111T/C* of the *CLOCK* gene is not associated with BMI however, data suggest that the minor allele of the *CLOCK 3111T/C* gene polymorphism may have a protective role in atherogenic lipid levels in women with a BMI greater than or equal to 25 kg/m^2^.

## 1. Introduction

The prevalence of being overweight and obesity is growing steadily in developed and developing countries [1]. It is known that being overweight can contribute to the development of diseases such as diabetes [2], cardiovascular diseases [3], asthma [4], cancer [5], and others. In this context, the task of finding predictors of being overweight does not lose its relevance.

Accumulated to date, some research results indicate that many metabolic and physiological functions are controlled by the circadian system, including one of its genes the *Circadian locomoter output cycles protein kaput* (*CLOCK*) gene. Its participation in the regulation of circadian rhythms is noted in the transcription–translation feedback loops and occurs through the dimerization of its protein product with the Brain muscle arnt-like (BMAL1) protein in the cell nucleus [6]. Currently, the single-nucleotide substitution in the 3′-untranslated region (UTR) of the *CLOCK* gene (*3111T/C*, *rs1801260*) is the most studied. Associations of this single-nucleotide polymorphism (SNP) were identified not only with the sleep–wake cycle [7,8,9,10], but also with body weight [11,12]. Therefore, it has been shown that individuals with a *C*-allele have lower body weight [11] and are less likely to be overweight [12]. Some studies have drawn opposite conclusions [13]. In a study examining the effect of diet on the lipid profile in overweight and obese patients, including those with metabolic syndrome and coronary heart disease, there were no differences in lipid profile parameters in groups with different *3111T/C* SNPs of their *CLOCK* gene [14,15,16]. It should be noted that the researchers did not differentiate their groups by gender, although it has been found that *3111T/C* SNP of the *CLOCK* gene is associated with the risk of overweight/obesity only in women [17,18].

It is known that being overweight and obesity occur in more than 60% of menopausal women [19]. This is due to involutional hormonal changes, primarily estrogen deficiency [20,21]. At the same time, given the above studies, it becomes clear that studies on the SNP *3111T/C* of the *CLOCK* gene and lipid profile association in this population cohort are needed. The results of studies by Galbete C et al. (2012) showed that the minor allele SNP *3111T/C* of the *CLOCK* gene could be associated with decreased overweight/obesity risk in elderly women [17]. A study involving girls showed *C*-allele and an increased body mass index (BMI) association [18]. Data on lipid profile parameters depending on the studied polymorphism genotype were not presented in either study. Given the multidirectional results of these studies, it is necessary to study the possible associations of SNP *3111T/C* of the *CLOCK* gene with the lipid profile separately in each age group. The high prevalence of obesity in menopausal women was the reason for choosing this critical period of life to study the effect of SNP *3111T/C* in the *CLOCK* gene on lipid outcomes. The obtained results can be used to prevent the development of lipid metabolism disorders.

The hypothesis of this study was a possible influence of *CLOCK* SNP on BMI and the lipid profile in menopause. To confirm this hypothesis, we carried out lipid profile comparative analysis in menopausal women with a BMI < 25 kg/m^2^ and those with a BMI ≥ 25 kg/m^2^ to reveal its interdependence with genotype regarding *3111T/C* SNP of the *CLOCK* gene.

## 2. Materials and Methods

### 2.1. Standard Protocol Approvals, Registrations, and Patient Consent

The study was carried out in the Federal State Public Scientific Institution “Scientific Centre for Family Health and Human Reproduction Problems” with the informed consent of the participants as provided for by the Ethical norms of the Declaration of Helsinki of the World Medical Association (2013) [22]. The research protocol was approved by the Committee on Biomedical Ethics of this Scientific Centre (protocol no. 8 dated 15 December 2016).

### 2.2. Subjects

This retrospective study involved 193 Caucasian peri- and postmenopausal women-volunteers aged from 45 to 60 years who were recruited through personal interviews. The research program was conducted and included the following methods: Clinical-anamnestic (medical history, physical examination, and gynecological examination), laboratory (molecular genetic testing and examination of lipid profile parameters), and statistical. Inclusion criteria for the perimenopausal group were as follows: Aged 45–55 years; oligomenorrhea or amenorrhea during the previous 12 months; mismatch of the structure and thickness of the endometrium corresponding to the first and the second phases of the menstrual cycle; and depletion of the follicular reserve of ovaries. Inclusion criteria for the postmenopausal group were as follows: Aged 56–60 years; amenorrhea for ≥12 months; a follicle-stimulating hormone level of >20 iU/mL, luteinizing hormone/follicle-stimulating hormone index < 1; thin non-functional endometrium, with endometrial echo thinner than 5 mm; and a lack of follicular reserve of ovaries. The exclusion criteria were as follows: Hormone replacement therapy; the use of lipid-lowering medication; and surgical menopause.

Women were categorized based on the results of clinical-anamnestic examination and according to the World Health Organization BMI ranges [1] as normal weight (18.5–24.9 kg/m^2^) (control), overweight (25.0–29.9 kg/m^2^), or obese (30.0 kg/m^2^ or above). Subjects that were overweight and had obesity were merged into the main group (those with BMI of ≥25 kg/m^2^). The basic characteristics of the groups are demonstrated in Table 1.

### 2.3. Methods and Elaborating on Methods

#### 2.3.1. Obesity-Related Parameters

The anthropometric measurements were performed in the morning, from 8:00 to 9:00 a.m. before blood sampling after an overnight fast beginning at 10:00 p.m. the previous night. Weight was measured to the nearest 0.1 kg using a balance scale, in light clothing, without shoes. Height was determined using a fixed wall stadiometer to the nearest 0.1 cm. The BMI was calculated as the weight in kilograms divided by the square of the height in meters.

#### 2.3.2. Collection of Material

Between 8.00 and 9.00 a.m., after 12 h of overnight fasting, venous blood was sampled from the cubital vein into two tubes with tripotassium ethylenediaminetetraacetic acid anticoagulant. Whole venous blood from the first tube served as the material for molecular genetic examination. Samples from the second tube were centrifuged for 10 min at 1.500× *g* at 4 °C and blood serum was used immediately for total cholesterol (TC), high-density lipoprotein cholesterol (HDL-C), and triacylglycerol (TG) determination.

#### 2.3.3. Molecular Genetic Examination

The study participants were genotyped according to the *3111T/C* SNP of their *CLOCK* gene (*rs1801260*). Subsequently, genomic DNA was extracted from the blood samples using the “AmpliPrime DNA-Sorb-B” reagent kit (“Nekstbio”, Moscow, Russia). The polymorphic marker was determined using polymerase chain reaction–restriction fragment length polymorphism technology on the “DT-Prime” (“DNA-Technology”, Moscow, Russia) amplifier using reagent sets for genotyping polymorphic markers (“TestGen”, Ulyanovsk, Russia). The *3111T/C* SNP of the *CLOCK* gene was amplified using forward (5′- TCC AGC AGT TTC ATG AGA TGC-3′) and reverse (5′-GAG GTC ATT TCA TAG CTG AGC-3′) primers. Amplification was carried out under the following conditions: 94 °C for 4 min, followed by five cycles of 95 °C for 30 s, 58 °C for 30 s, and 72 °C for 60 s; thereafter, 30 cycles of 95 °C for 30 s, 55 °C for 30 s, and 72 °C for 60 s. The SNP was identified by restriction of the 221bp PCR-fragment with the restriction enzyme Bsp1286I at 37 °C for 5 h and the *C*-allele was cut by Bsp1286I. The resulting fragments were visualized on a 3% agarose gel under ultraviolet light after ethidium bromide staining. The electrophoresis results were recorded and documented using the GelDoc computerized gel documentation system.

#### 2.3.4. Plasma Lipid Determination

TC, HDL-C, and TG levels were measured photometrically using PLIVA-Lachema kits (Brno, Czech Republic) on a BTS-330 automatic analyzer (Barcelona, Spain). The low-density lipoprotein cholesterol (LDL-C) and very-low-density lipoprotein cholesterol (VLDL-C) levels in serum were estimated mathematically using the Friedewald formula [23]. This formula was used because the TG levels were less than 4.5 mmol/L. LDL-C was calculated as TC–HDL-C–VLDL-C, and VLDL-C was calculated as TG/2.2 because the unit is mmol/L.

#### 2.3.5. Statistics

The obtained data were processed in STATISTICA 10 (Stat-Soft Inc, Tulsa, OK, USA). A power calculation was not performed for this retrospective genotyping analysis. To determine the proximity to the normal law of distribution of quantitative signs, the visual-graphic method and the Kolmogorov–Smirnov test were used. The Fisher test was used to determine the equality of the general variances. Lipid profile parameters were characterized by a non-normal distribution therefore, the assessment of these parameters’ differences between the studied groups was carried out by a nonparametric method of statistical analysis using the Mann–Whitney *U*-test. Lipid profile data are presented as median [quartile 1 (Q1); quartile 3 (Q3)]. Data on age, weight, height, and BMI followed a normal distribution and were analyzed using the parametric Student *t*-test. These data are presented as arithmetic mean ± standard deviation. The distribution of the genotypes of the investigated polymorphism was tested for compliance with the Hardy–Weinberg law. Comparisons of the frequencies of alleles and genotypes within and between the targeted groups were carried out using the Pearson χ^2^ test with Yates correction for continuity if the value of at least one cell of the conjugation table was less than 5. The genotypes and the overweight risk association were assessed by determining the odds ratios. Discriminant analysis to select the most informative parameters underlying the division of groups with different genotypes was used. All the differences were considered statistically significant at *p* < 0.05.

## 3. Results

A comparative analysis of the distribution of alleles of the *CLOCK 3111T/C* gene polymorphic marker was conducted for the studied groups of women (Table 2). In both groups, the distribution of the genotypes corresponded to the Hardy–Weinberg distribution law (*p* > 0.05). In both groups, normal weight and overweight women, the *3111T*-allele and the *TT*-genotype were more prevalent. There were no statistically significant differences in the frequency of occurrence of the genotypes and alleles of the studied SNP between the compared groups.

When calculating the odds ratios of the risk of overweight depending on the genotype, no association was found (Table 3).

The results of the discriminant analysis showed that the most informative lipid profile parameters distinguishing the group with the *TT*-genotype from the group with the *TC-*, *CC*-genotypes were TC (F = 14.71; *p* = 0.000) and LDL-C (F = 27.22; *p* = 0.000). The square of the Mahalonobis distance was 1.23 (*p* = 0.000). Recalculation of the parameters’ informativeness as a percentage showed the contribution of TC to be 28.5% and that of LDL-C to be 44%.

Table 4 summarizes the lipid profile parameters in women with/without a BMI of ≥25 kg/m^2^ differentiated as carriers of different genotypes of *3111T/C* SNP of the *CLOCK* gene. Given the small number of women carrying the *CC*-genotype of the *CLOCK 3111T/C* gene, the carriers of the *CC*-genotype and *TC*-genotype were combined into one group as carriers of the minor *3111C*-allele. There were no statistically significant differences in the lipid profile parameter levels in the group with normal-weight carriers of different SNP genotypes (the *TT*-genotype and the *TC-, CC*-genotypes). When comparing the given women with a BMI ≥ 25 kg/m^2^, significantly higher TC and LDL-C levels were detected in the carriers of the *TT*-genotype as compared to the carriers of the minor *3111C*-allele (*p* < 0.05). A comparative analysis of the lipid profile between groups with a normal weight and were overweight showed higher levels of TC, TG, and LDL-C and lower HDL-C levels in women with a BMI ≥ 25 kg/m^2^ who are carriers of the *TT*-genotype, as compared to the group with normal weight and the *TT*-genotype (*p* < 0.05). The carriers of the minor allele with a BMI ≥ 25 kg/m^2^ had higher TC, TG, and LDL-C levels as compared to the group with normal weight and the *3111C*-allele (*p* < 0.05).

## 4. Discussion

It is currently supposed that being overweight may be associated with *3111T/C* SNP of the *CLOCK* gene, but the results of studies on the matter are ambiguous [11,12,13,14,15,16,17,18]. Here, we found no differences in the prevalence of genotypes and alleles of *3111T/C* SNP of the *CLOCK* gene in women that had a normal weight or were overweight. Our data are consistent with the results of a study involving elder women [17]. In addition, similar data were obtained for a sample including both women and men of reproductive age however, the authors then compared groups with a BMI of ≥40 kg/m^2^ and BMI of <40 kg/m^2^ and found an association of the minor allele with class III obesity [13]. Using these results, we conducted a similar analysis and did not find differences in BMI between groups of different genotypes with being overweight. In addition, among the participants in our study with the *CC*-genotype, there were no women with a BMI of >40 kg/m^2^. A calculation of the odds ratios of overweight realization in groups with the *TT*-genotype and *TC-*, *CC*-genotypes also showed no association of genotypes with being overweight. Based on our results, it can be assumed that the SNP *3111T/C* of the *CLOCK* gene is not associated with being overweight in menopausal women.

Using multivariate discriminant analysis, we tried to determine the most informative lipid parameters according to which groups with different genotypes differ, but without dividing by BMI. It turned out that the greatest contribution to the difference between the groups was made by LDL-C and TC. To understand whether BMI affects lipid outcomes across genotypes, we analyzed groups with a normal BMI and those who were overweight. The results obtained indicate increased LDL-C and TC levels in women with the *TT*-genotype in the overweight group. Our results are, to some extent, consistent with the results of a study by Tsuzaki K. et al. (2010), who showed that in *TT*-homozygotes, the area of sdLDL-C was greater compared with that in carriers of the *C*-allele. Their group was not divided according to gender or age, including participants aged 24 to 88 [24]. In turn, the results of some studies on a potential association of SNP *3111T/C* of the *CLOCK* gene and obesity showed that gender and age are important factors influencing the outcome. In a recent study involving schoolchildren, an association of the minor allele with being overweight was found only in girls [18]. Opposite results, which are consistent with our data, were obtained with elder women [17]. A possible reason for these differences may be different sex hormone levels.

Many studies have shown a regulatory role for estradiol in lipid metabolism and food intake [25,26]. The results of a recent experimental study showed that the estradiol synthesis in granulosa cells is regulated by melatonin. At the same time, it was shown that low melatonin concentrations have an effect on 89 differentially expressed genes associated with steroid hormone synthesis, cell proliferation and cell cycle regulation, positive regulation of the nitrogen compound metabolic process, oxidoreductase activity, and anti-apoptosis regulation [27].

We currently know about age-related decreases in melatonin concentration, indicating a decrease in the melatonin-forming function of the pineal gland, which is a consequence of functional changes in the pineal gland and in other links of the circadian system of the body during physiological aging [28]. It has been shown that melatonin rhythms in menopausal Caucasian women are associated with *3111T/C* SNP of the *CLOCK* gene. A shift of the melatonin peak to the early morning hours was recorded in carriers with the major allele [10], which is considered a possible cause of the development of oxidative stress in these women [29]. In turn, oxidative stress leads to epigenetic changes that involve chromatin remodeling via alterations in transcriptional regulators with a modification of histones leading to metabolic disorders with neurodegeneration [30].

One of the genes associated with the circadian clock is *SIRT 1*. It is possible that *SIRT 1* defects lead to suprachiasmatic nucleus in the hypothalamus, defects that are related to peripheral circadian clock dyssynchrony and changes in melatonin levels [31]. It has also been shown that *SIRT 1* regulates food intake and body weight [32]. The results of an experiment on mice aiming to study the effect of melatonin on the lipid profile and the development of obesity demonstrated that melatonin supplementation attenuated serum TG, TC, and LDL-C levels and prevented body mass gain through a decreased lipogenesis rate and increased lipolytic capacity in white adipocytes, with a concomitant increment in oxygen consumption and *PGC1A* and *PRDM16* expression [33]. Taking these results into account, we suggest that the TC and LDL-C level increases in overweight women with *3111T/C* SNP of the *CLOCK* gene major allele in our study may be associated with melatonin secretion changes.

The results of a recent study on the functional role of *3111T/C* polymorphism of the *CLOCK* gene showed that the *C*-allele, as compared to the *T*-allele, leads to higher *CLOCK* mRNA levels and a high expression of *PER2*—a CLOCK transcriptional target—thereby changing the other clock molecules’ network and circadian rhythmicity. It is assumed that these changes may be due to different mechanisms, including the binding sites of miRNA-182 in the 3′-UTR of the *CLOCK* gene [34].

Among the studies on this topic, we found a series of investigations indicating lower adiponectin and high ghrelin levels in overweight and obese people with *3111T/C* SNP of the *CLOCK* gene’s minor allele [35,36]. It is known that adiponectin has an anti-atherogenic effect [37] and ghrelin is an important factor linking the central nervous system with peripheral tissues that regulate lipid metabolism [38].

## 5. Conclusions

In conclusion, the present results showed that menopausal women with a BMI ≥ 25 kg/m^2^ and the TT-genotype had higher TC and LDL-C levels when compared to carriers of the minor allele, which may play a protective role in the atherogenic lipid levels in these women. There are some limitations to our study. First of all, the number of women in the groups was limited. Studies with larger cohorts may find lipid profile changes in menopausal women with the homozygous *CC*-genotype. Secondly, our study included only menopausal women. Similar studies with women of reproductive age may reveal the influence of age on an association of *3111T/C* SNP of the *CLOCK* gene and lipid metabolism. Thirdly, the study did not include estrogen data. Determination of female sex hormones may demonstrate an association of the SNP and estrogens. In addition, the contribution of other components of the circadian oscillator to genetic susceptibility to overweight should be investigated.

## Figures and Tables

**Table 1:** Profile of women with different body mass index (BMI).

Characteristics	BMI < 25, *n* = 42	BMI ≥ 25, *n* = 151
Age, year	52 ± 4.74	53 ± 5.15
Height, m	1.62 ± 0.06	1.61 ± 0.07
Weight, kg	59.9 ± 6.54	81.1 ± 13.21
BMI, kg/m^2^	23.1 ± 1.51	31.2 ± 4.95 *
Perimenopause, *n* (%)	20 (47.62)	61 (40.4)
Postmenopause, *n* (%)	22 (52.38)	90 (59.6)

*–*p*-value < 0.05 as compared to control.

**Table 2:** The frequencies of the *Circadian locomoter output cycles protein kaput (CLOCK) 3111T/C* (*rs 1801260*) gene polymorphism genotypes and alleles in the groups.

Group	Genotypes, *n* (%)			Alleles		Compliance with the Hardy–Weinberg Law			
						Expected Genotype Frequency (%)			*p*-Value
	*3111T/T*	*3111T/C*	*3111C/C*	*3111T*	*3111C*	*3111T/T*	*3111T/C*	*3111C/C*	
BMI < 25 kg/m^2^	22 (52.4)	16 (38.1)	4 (9.5)	0.71	0.29	51.05	40.8	8.15	>0.05
BMI ≥ 25 kg/m^2^	76 (50.3)	56 (37.1)	19(12.6)	0.69	0.31	47.4	42.89	9.7	>0.05
	χ2 = 0.294; df = 2; *p* = 0.864			χ2 = 0.202; df = 1; *p* = 0.654					

**Table 3:** The odds ratios of the risk of being overweight depending on the genotype.

Overweight Risk Factor	OR (95% CI)	*p*-Level
*TT*-genotype	0.92 (0.47–1.83)	>0.05
*TC-*, *CC*-genotypes	1.09 (0.55–2.15)	>0.05

**Table 4:** BMI and lipid profile parameters in women who are carriers of different *CLOCK 3111T/C* gene polymorphisms (*rs 1801260*).

Parameters	BMI < 25 kg/m^2^		BMI ≥ 25 kg/m^2^		*p*-Value
	*3111T/T*	*3111T/C + 3111C/C*	*3111T/T*	*3111T/C + 3111C/C*	
	*n* = 22	*n* = 20	*n* = 76	*n* = 75	
BMI (kg/m^2^)	23.2 ± 1.66	23 ± 1.35	31.1 ± 4.46	31.2 ± 5.44	*p ^a,c^* = 0.009 *p ^b,d^* = 0.001
TC, (mmol/L)	4.25 [3.70–5.37]	4.39 [3.99–4.53]	5.29 [4.72–6.02]	5.18 [4.09–5.91]	*p ^a,c^* = 0.013 *p ^c,d^* = 0.029 *p ^b,d^* = 0.033
TG, (mmol/L)	0.67 [0.54–0.99]	0.94 [0.77–1.12]	0.98 [0.74–1.23]	1.01 [0.68–1.39]	*p ^a,c^* = 0.010 *p ^b,d^* = 0.025
HDL-C, (mmol/L)	1.34 [1.23–1.58]	1.19 [1.05–1.36]	1.15 [0.92–1.34]	1.24 [1.02–1.40]	*p ^a,c^* = 0.018
LDL-C, (mmol/L)	2.60 [2.14–3.27]	2.69 [2.33–2.92]	3.87 [3.10–4.29]	3.51 [2.43–4.06]	*p ^a,c^* = 0.003 *p ^c,d^* = 0.012 *p ^b,d^* = 0.007
VLDL-C, (mmol/L)	0.30 [0.24–0.45]	0.43 [0.35–0.58]	0.45 [0.34–0.56]	0.46 [0.31–0.64]	NS

NS—not statistically significant, *p* > 0.05. Groups: *a*—*3111T/T*, BMI < 25 kg/m^2^; *b*—*3111T/C+ 3111C/C*, BMI < 25 kg/m^2^; *c*—*3111T/T*, BMI ≥ 25 kg/m^2^; *d*—*3111T/C + 3111C/C*, BMI ≥ 25 kg/m^2^. TC–total cholesterol; HDL-C–high-density lipoprotein cholesterol; TG–triacylglycerol; LDL-C–low-density lipoprotein cholesterol; VLDL-C–very-low-density lipoprotein cholesterol.

## Data Availability

Restrictions apply to the availability of these data.
